# Evaluating Cultural Ecosystem Services of Urban Residential Green Spaces From the Perspective of Residents' Satisfaction With Green Space

**DOI:** 10.3389/fpubh.2020.00226

**Published:** 2020-07-17

**Authors:** Qizheng Mao, Luyu Wang, Qinghai Guo, Yuanzheng Li, Min Liu, Guanghua Xu

**Affiliations:** ^1^Department of Resource and Environmental Science, Henan University of Economics and Law, Zhengzhou, China; ^2^School of Civil Engineering and Architecture, Zhejiang Sci-Tech University, Hangzhou, China; ^3^Jiyang College, Zhejiang Agricultural & Forestry University, Zhuji, China

**Keywords:** cultural ecosystem services, residential districts, green space, satisfaction, physical environment

## Abstract

Green spaces in residential areas provide multiple cultural ecosystem services (CES), which can contribute to human health by increasing the frequency of residents' visits. We evaluated the CES of residential green spaces by assessing residents' satisfaction with these spaces in the city of Zhengzhou, China. The data reveal the supply capacity of CES in residential green spaces: the results suggest that the level of recreational services is low, whereas the residents' satisfaction with the sense of place and neighborhood relations is high. The lower the frequency of residents who visit a park outside the residential area, the higher the satisfaction with the CES. This suggests that residential green spaces can effectively compensate for the lack of nearby parks owing to their proximity to residents' living quarters. The CES in residential communities increased as vegetation coverage increased, indicating that natural vegetation is a source of CES. In addition, the results showed that residents' perceptions of plant decoration, landscape patterns, and management and infrastructure in particular can effectively improve the level of CES, and this could compensate for CES that have shrunk owing to low green space coverage. This study has practical significance and value for the planning and design of residential green spaces, offering suggestions for urban landscape planners and decision makers. Future research should combine the residents' perception of demand and supply of CES and should clarify the gap and trade-off between them.

## Introduction

The ecosystem services of urban green spaces can be defined as services that improve the welfare of urban residents who enjoy green spaces (1). These services include support, regulation, supply, and cultural ecosystem services (CES) (2). CES can be provided by green spaces for leisure, tourism, cultural education, aesthetic appreciation, and spiritual needs (3), all of which account for a large proportion of the ecosystem services in an urban green space (4). Residents' physical and mental health, especially social belonging, group identity, and social integration, are closely related to environmental services (5). In recent years, CES have become a trending topic in urban ecosystem services research. However, compared with other types of ecosystem services, research on CES is still in its infancy because its intangible characteristics are difficult to quantify.

Currently, several researchers are exploring various means to study the CES of urban green spaces (6–13). Survey questionnaires, the most commonly used evaluation method, can be direct or indirect. Direct methods includes face-to-face (5, 6), email (7, 8), or network (9, 10) surveys, which evaluate CES according to residents' visit frequency to green spaces, activity types, and perceptions of CES. Indirect methods capture pictures of the target place and then invite residents to give scores to the CES reflected by the different green space landscapes or land use types in the pictures (11, 12). Although face-to-face questionnaires are an effective way to evaluate urban ecosystem services, they are also difficult, mainly because of the high cost (8).

Previous studies have quantified CES in green areas from the residents' perceptions (10, 13), producing CES scores for subjective cognition and distinguishing the importance of different CES (9, 14–16). Moreover, by detecting the population's perspective on need, this research has described the demand for CES. The trade-off between the demand and supply of CES is also a popular research topic that can provide constructive suggestions for urban management and planning. CES supply in urban areas is characterized by the spatial distribution and physical attributes of urban green spaces, such as the amount, size, type, water bodies, facilities, and biodiversity (17). Residents' satisfaction with their surrounding physical environment is commonly used in studies concerning the well-being of humans (18, 19); such parameters objectively portray the status (i.e., positive or negative) of an urban environment from the residents' perspective. Therefore, the evaluation of residents' satisfaction with CES can directly present the supply capacity of CES, which will facilitate any adjustment of the physical characteristics of green spaces. However, few studies have assessed CES from the perspective of residents' satisfaction with green spaces.

Urban residents around the world express a desire for contact with nature and one another, including attractive environments, recreational and play areas, privacy, active roles in the design of the community, and a sense of community identity (20). A positive correlation exists between human health and urban green spaces (21–23). Urban green spaces refer to natural vegetation in cities, including highly artificial green spaces, such as roadside green parks, residential green space, and natural woodland, which provide a variety of ecosystem services, especially recreational and entertainment areas with CES. Numerous studies have focused on the CES of urban parks, forests, wetlands, and other popular green spaces (6, 24, 25), whereas residential areas have received minimal attention. With the rapid expansion of cities and resultant growth of the urban population, as well as the limited natural vegetation in cities, urban residential areas are becoming gradually dominated by environments that have a high population density and a low green space density. In particular, the green areas of urban residential areas in developing countries are often correlated with real estate prices (26), which leads directly to the prevalence of urban human settlements and environmental inequity (27). Scholars have therefore pointed out that the remaining green spaces should make up for the shortage of other green land types, such as roadside and residential green spaces (9). The distance between urban green spaces (e.g., parks) and residents determines the use frequency of these spaces (10, 28, 29). Residential green spaces are the most common and frequently used land types and have multiple ecosystem functions and services (e.g., biodiversity protection, climatic adjustment, energy saving, and recreation). Therefore, evaluating and exploring the CES characteristics of green areas in high-density residential areas can provide valuable references for urban ecosystem research. As an essential component of urban green spaces, residential green spaces are characterized particularly by high fragmentation and heterogeneity, and huge differences exist among residential districts, which are correlated with various landscape planners, property managers, and residents with different socioeconomic status. Hence, research on CES in urban residential green spaces is difficult to conduct. Many studies have reported that the design of urban landscapes greatly influences the well-being and behavior of users and nearby inhabitants (18, 30). CES in high-density residential areas are thus more important than the regulative and supporting services of green spaces. Moreover, magnifying the CES in limited spaces is significant. Investigating and assessing CES in residential areas can further enrich the theories and practices of ecosystem services in urban green spaces.

The influencing factors of CES in urban green spaces are a key research topic, which could provide important and practical information for the planning and management of urban green spaces. The CES in urban green spaces are often related to residents' socioeconomic status (e.g., age, income level, marriage, and profession) (14, 31–34). Specifically, residents' socioeconomic status is closely related to the subjectivity and intangibility of CES. In addition, urban morphologies and land use may affect CES in large-scale green spaces. For instance, CES in wetlands are better than in other land types (9, 35). The quality and quantity of green space landscapes are the important influencing factors of CES (36), along with green space size (37), green space accessibility (38), natural properties of green spaces (9, 14, 39), and species composition and biodiversity (40, 41). Studies have found that cleanliness and proper management (9), as well as infrastructure (42), contribute greatly to improvement of CES in green spaces. Green spaces in residential areas offer various CES, such as walking, exercise, aesthetic appreciation, neighborhood exchanges, stress-relieving activities, and activities that foster a good mood. Hence, exploring the influencing factors of CES in residential green spaces should provide important practical guidance for landscape planning and the design of green spaces in residential areas.

Previous studies have focused on residents' satisfaction with their living environment (10, 14, 33, 42). Physical and natural environments exert significant effects on residential satisfaction with the aspects of the natural environment, convenient transportation, environmental health, urban security, the convenience of public facilities, and the sociocultural environment. However, the following questions remain: (1) How does the natural environment affect the human perspective of the residential environment? (2) How satisfied are people with urban green spaces in residential areas? (3) What is the current level of CES in the residential green spaces of high-density communities? (4) Is the vegetation coverage of green spaces a major factor that affects CES? (5) What physical environment of residential green spaces contributes the most to CES? These questions can be answered by evaluation of CES and exploration of the possible determinant factors of CES in residential green spaces.

This study aimed to evaluate the level of CES in green spaces of residential areas by examining the satisfaction of residents and to explore the possible factors affecting the function of residential green spaces (e.g., coverage of green space in the residential areas, social factors, residents' use of green spaces, and management) and the key issues that should be addressed.

## Materials and Methods

### Study Area

A total of 40 residential communities in Jinshui District, Zhengzhou City, Henan Province, China, comprised the study area. Zhengzhou, the capital of Henan Province, is Henan's largest and most populated city and has an area of 7,446 km^2^ and 9.88 million inhabitants as at 2018. Most housing estates in the Jinshui District are relatively mature, containing not only medium vegetation coverage but also housing built before 2010. We assumed that these housing estates would provide a stable and objective level of CES.

Among the 40 major cities in China, Zhengzhou has the highest population density at 15,000 people/km^2^, and the urban land conflict is most prominent. The current urbanization rate of Zhengzhou is 78.2%, ranking 30th in China, and the urbanization process in this city is advancing rapidly. Many real estate resources have been built, but their overall quality is low. In particular, Zhengzhou has low green space coverage and property management with different levels. Gaps between the living environment of Zhengzhou's urban residents and other first-tier cities in China (e.g., Beijing, Shanghai, Shenzhen, and Guangzhou) are evident; that is, Zhengzhou lags behind in terms of urbanization and economic development. Evaluating the current supply capacity of the CES of residential areas can provide a scientific reference for improving the living environment and well-being of Zhengzhou residents.

**Figure 1:**
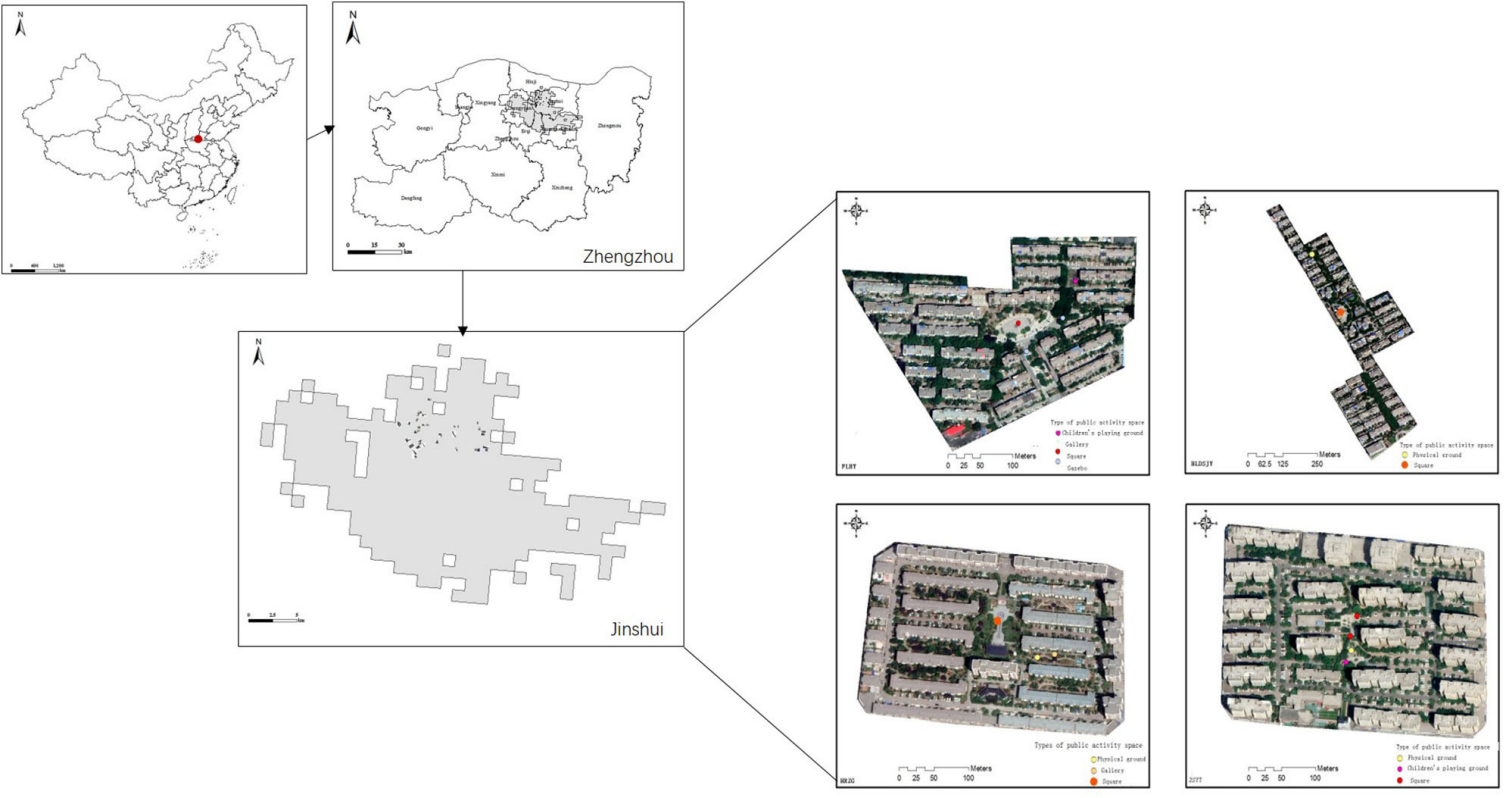
The geographical locations of the selected 40 residential communities.

Zhengzhou is divided into six administrative districts and seven provincial direct counties. Jinshui District is one of the most economically developed urban areas in the province, with a total area of 135.3 km^2^ and a population of 1.402 million. Jinshui District is the area with the largest population and the most developed economy in Zhengzhou (Figure 1). Compared with the other districts' residential areas, the real estate development area in Jinshui District is the largest, earliest, and most mature. The residential projects developed in the Jinshui District are composed of 50% ordinary housing and almost 40% villas and affordable housing. These patterns are closely related to the comprehensive functions undertaken by the Jinshui District and the spatial development strategy of “Northward and Eastward Expansion.” The dominant type of residential area in the Jinshui District is the reason why this area was chosen for the case study (43).

Jinshui District is also the most active urban expansion area in Zhengzhou. The demand for residential areas from the urban population is increasing, leading to development of high-density residential areas in the region at the expense of green spaces and a reduction of ecosystem services.

### Classification and Evaluation Indicators of Cultural Ecosystem Services in Green Spaces in Residential Areas

CES can be classified under the non-material benefits provided by ecosystems (44). CES in urban green spaces are globally categorized into seven types: aesthetic information, recreation, cultural heritage, education, social relations, health, and spiritual/religious values (2, 45, 46). In residential areas, CES could be defined as opportunities for residents to enjoy recreational activities, aesthetic appreciation, social contact with neighbors, and stress-relieving activities and to strengthen the sense of belonging. CES in residential green spaces are divided into five types: recreation, aesthetics, social relations, a sense of belonging, and spiritual demand (Table 1). Recreational services refer to various recreational activities available in the residential green space for the residents, including exercise, walking, dog walking, childcare, and running. Recreational services are evaluated by measuring the frequency and duration of residents' participation and their satisfaction. Aesthetic services are residents' aesthetic perceptions of the overall landscape and plant collocation in residential green spaces, and they are evaluated by the residents' overall satisfaction with the aesthetics of the above two factors. Social relations services provide residents with opportunities to communicate with neighbors and release emotional stress. This service is evaluated through the communication frequency of residents with family members, friends, or neighbors, as well as their satisfaction with neighborhood relations. The evaluation index for the sense of belonging involves residents' satisfaction with respect to how welcoming and nurturing the environment is. Finally, spiritual services involve spiritual experience and spiritual release in residential green spaces. The evaluation indexes for spiritual services include satisfaction with pressure relief features and the quietness of the environment. The quality of urban green spaces is widely evaluated by residents' satisfaction with various functions (15, 19, 34). Therefore, in our study, we applied the satisfaction with CES to identify the level of CES.

**Table 1:** Selected CES and their indicators.

**CES**	**Indicators**
Recreation	Visiting types, visiting frequency, residence time, and satisfactory recreation
Aesthetic value	Satisfactory aesthetic of the green space landscapes and plant collocation
Social relationship	Chat frequency, satisfactory neighborhood relation
Sense of belonging	Satisfactory sense of belonging
Spiritual value	Stress relieving features, Quietness of the environment

### Data Collection

A face-to-face survey was used to explore the attitudes of residents in Zhengzhou City toward the different types of CES in the residential green spaces. To ensure the adequacy of the sample size, as well as the authenticity of the questionnaire, we selected 40 sites from the 215 residential communities in Jinshui. These sites, all of which were built after 2000, have at least 600 households. The area of the 40 residential estates ranged from 0.38 to 33 hm^2^, and the vegetation cover ratio was between 13 and 58%. In China, a residential community is the smallest residential unit within a limited space (Figure 1). Each residential community has unique characteristics, such as vegetation coverage, water bodies, public activity spaces, management, infrastructure, vegetation structure, and plant species. Moreover, different communities are relatively independent and closed. Therefore, our study investigated residents' overall satisfaction with different types of CES and the use of green spaces in these sites.

To truly express the impact of green spaces on residents' activities, the data were collected on weekends and official holidays from June 2017 to August 2018. The survey was conducted between 09:00 and 19:00. Our interviewees were mainly residents who are inactive in the green spaces of the community. A household survey was used as a supplement to ensure a sufficient sample size. A total of 4,519 respondents were interviewed, with between 93 and 135 interviewees from each of the 40 communities. First, residents' use of green spaces and satisfaction with different types of CES were collected to evaluate the cultural service levels of the green spaces. Then, the residents' satisfaction with green space management and infrastructure was investigated. Finally, the most satisfying and unsatisfying factors with regard to the residential green spaces were collected, covering management, water services, public activity spaces, green coverage, facilities, plant collocation, and the landscape pattern of the green spaces, to analyze the subjective physical environment of the residential areas by investigating the influencing factors of CES in the residential green spaces. The collected physical environment indicators included real vegetation coverage, the number of public activity spaces, including the existence of water bodies, and the management level. With the use of Google's high-definition imagery, object-oriented automatic classification was applied to extract the real vegetation coverage, which refers to the vertical projection area of vegetation (including leaves, stems, and branches) on the ground as a percentage of the total area of the residential area. The number of public activity spaces (e.g., squares, water bodies, children's play facilities, gazebos, promenades, and places for physical exercise) was obtained during the survey of residents' satisfaction with CES. A total of 40 residential areas were categorized into two groups: residential areas with water body settings and those without. The management of residential green spaces was subjectively divided into three levels: good, medium, and poor.

### Data Analysis

All data aggregation and statistical analyses were conducted in Microsoft Excel and SPSS v21. First, we analyzed the descriptive statistics to explore the socioeconomic characteristics of the respondents (gender, age, income, education, and the use of residential green spaces) and their ways of using green areas (Table 2). The level of respondents' satisfaction with the various CES in the residential green spaces was scored 1–10, with 1 indicating poor satisfaction and 10 maximum. The overall satisfaction with CES was calculated as an average of satisfaction with seven types of CES, including recreation, the aesthetics of the green space landscapes and plant collocation, neighborhood relations, the sense of place, stress-relieving features, and the quietness of the environment. Pearson's correlation analysis was used to investigate the relationship among the various CES in the residential green spaces (Pearson's coefficient) (Table 3). Linear regression analyses were applied to test the possible variables affecting the level of CES. The analysis process was as follows (47): single-factor results were derived from a univariate linear regression model that included a single variable (Table 4), and significant variables emerging from the single-factor models (*p* < 0.05) were then included in subsequent multivariate linear models (i.e., social–economic attributes, green spaces' use, frequency of visits to parks outside the residential area, and the subjective and objective physical environmental variables) (Table 5), which were examined in a series of backward stepwise elimination procedures. The final multivariate linear regression models included all the demographic variables and the successive inclusion of significant variables from the socioeconomic factors, use of green space, and the physical environmental variables selected by the backward stepwise procedures (*p* < 0.05).

**Table 2:** Respondents' demographic characteristics and use of residential green spaces.

**Characteristic**	**Frequency**	**Percentage (%)**
**Gender**		
Male	2,247	49.72
Female	2,272	50.28
**Length of study**		
<1 year	663	14.68
1–3 years	1,105	24.45
3–5 years	1,019	22.54
>5 year	1,815	40.16
**Age group**		
<20	773	17.1
21–29	890	19.70
30–39	1,101	24.36
40–49	686	15.17
50–59	455	10.07
>60	615	13.6
**Education**		
Junior high school or lower	1,253	27.73
High school	816	18.05
Junior college	963	21.3
Undergraduate	1,290	28.54
Post-graduate	198	4.38
**Income(RMB)**		
No income	1,428	31.6
1,000–3,000	700	15.5
3,000–5,000	1,322	29.25
5,000–10,000	838	18.55
>10,000	230	5.1
**Visiting frequency to parks**		
Everyday	464	10.26
At least three times a week	1,101	24.37
At least three times a month	985	21.79
Occasional	1,969	43.58
**Visiting frequency to residential green spaces**		
Everyday	1,779	39.36
At least three times a week	1,395	30.87
At least three times a month	528	11.68
Occasional	817	18.07
**Residence time(%)**		
Half an hour	1,991	44.05
1–2 h	1,701	37.65
Never	383	8.48
3 h	267	5.91
>3 h	175	3.87
**Chatting frequency(%)**		
Everyday	1,263	27.95
At least three times a week	1,388	30.71
At least three times a month	523	11.58
Occasional	776	17.18
Never	568	12.56

**Table 3:** Pearson's correlation coefficient of the different CES.

	**Recreation**	**Aesthetic of the landscape**	**Aesthetic of plant collocation**	**Quietness**	**Neighborhood relation**	**Stress relieving**	**Sense of belonging**
Recreation	1						
Aesthetic of the landscape	0.922^**^	1					
Aesthetic of plant collocation	0.924^**^	0.990^**^	1				
Quietness	0.931^**^	0.941^**^	0.944^**^	1			
Neighborhood relation	0.606^**^	0.538^**^	0.544^**^	0.599^**^	1		
Stress relieving features	0.920^**^	0.934^**^	0.930^**^	0.937^**^	0.689^**^	1	
Sense of belonging	0.914^**^	0.875^**^	0.889^**^	0.932^**^	0.754^**^	0.938^**^	1

** *p < 0.01*.

**Table 4:** Relationship between residents' satisfaction on different CES and demographic characteristics, use frequency of green spaces and variables of physical environment with the univariate linear regression analysis.

**Variables**	**Recreational**	**Aesthetic**	**Quietness**	**Neighborhood relation**	**Stress relieving**	**Sense of belonging**	**The total level of CES**
	**B (P)**	**B (P)**	**B (P)**	**B (P)**	**B (P)**	**B (P)**	**B(P)**
**RESIDENTS 'DEMOGRAPHIC CHARACTERISTICS (%)**							
**Age group**							
21–29 years	−0.47 (0.02)	0.06 (0.01)	0.05 (0.021)	−0.04 (0.004)	0.05 (0.009)	0.06 (0.002)	−0.05 (0.007)
50–59	0.05 (0.23)	0.09 (0.1)	0.08 (0.123)	0.06 (0.029)	0.08 (0.07)	0.09 (0.046)	0.07 (0.08)
>60	0.04 (0.151)	0.05 (0.117)	0.06 (0.041)	0.03 (0.07)	0.04 (0.103)	0.05 (0.042)	0.05 (0.07)
**Income (RMB)**							
1,000–3,000	−0.06 (0.08)	−0.08 (0.029)	−0.05 (0.218)	0.003 (0.895)	−0.05 (0.134)	−0.04 (0.254)	−0.05 (0.117)
**RESIDENTIAL GREEN SPACES “USE”**							
**Visiting frequency**							
At least three times a month	−0.004 (0.893)	0.02 (0.603)	0.01 (0.755)	−0.05 (0.021)	0.002 (0.953)	−0.017 (0.598)	−0.004 (0.889)
**Residence time (%)**							
Never	−0.074 (0.059)	−0.11 (0.02)	−0.08 (0.073)	−0.005 (0.854)	−0.05 (0.149)	−0.06 (0.148)	−0.06 (0.087)
1–2 h	0.04 (0.01)	0.04 (0.03)	0.04 (0.032)	0.02 (0.051)	0.04 (0.007)	0.03 (0.035)	0.037 (0.015)
**Chatting frequency (%)**							
At least three times a month	−0.009 (0.765)	0.002 (0.951)	−0.01 (0.699)	−0.04 (0.023)	−0.008 (0.756)	−0.03 (0.343)	−0.01 (0.617)
**VISITING FREQUENCY TO PARKS OUTSIDE (%)**							
At least three times a week	−0.03 (0.14)	−0.042 (0.078)	−0.04 (0.092)	−0.006 (0.645)	−0.04 (0.034)	−0.02 (0.246)	−0.03 (0.09)
At least three times a month	−0.04 (0.078)	−0.03 (0.197)	−0.04 (0.088)	−0.04 (0.004)	−0.04 (0.066)	−0.04 (0.056)	0.04 (0.062)
Occasional	0.03 (0.018)	0.03 (0.02)	0.03 (0.013)	0.01 (0.134)	0.03 (0.015)	0.02 (0.058)	0.026 (0.015)
**PHYSICAL ENVIRONMENTAL VARIABLES (OBJECTIVE)**							
Vegetation coverage ratio (%)	0.04 (0.02)	0.06 (0.002)	0.06 (0.001)	0.03 (0.001)	0.05 (0.003)	0.05 (0.002)	0.047 (0.003)
Level of management	0.68 (<0.001)	0.90 (<0.001)	0.76 (<0.001)	0.75 (<0.001)	0.61 (<0.001)	0.6 (<0.001)	0.653 (<0.001)
Number of spaces for public activities	0.09 (0.007)	0.13 (0.001)	0.11 (0.005)	0.04 (0.059)	0.10 (0.003)	0.1 (0.002)	0.098 (0.002)
Existence of water body	0.64 (0.036)	0.90 (0.011)	0.70 (0.045)	0.44 (0.021)	0.70 (0.02)	0.694 (0.02)	0.678 (0.016)
**PHYSICAL ENVIRONMENTAL VARIABLES (SUBJECTIVE)-PROPORTION OF RESIDENTS WHO ARE SATISFIED WITH PHYSICAL ENVIRONMENT (%)**							
Plant decoration	0.02 (0.017)	0.09 (<0.001)	0.08 (<0.001)	0.03 (0.022)	0.07 (<0.001)	0.04 (<0.001)	0.065 (<0.001)
Coverage of greenspaces	0.05 (0.012)	0.07 (<0.001)	0.06 (<0.001)	0.02 (0.035)	0.05 (<0.001)	0.045 (<0.001)	0.049 (<0.001)
Waterbody	0.05 (0.024)	0.05 (0.053)	0.06 (0.028)	0.002 (0.876)	0.03 (0.156)	−0.04 (0.017)	0.038 (0.069)
Space for public activities	−0.04 (0.056)	−0.07 (0.008)	−0.06 (0.013)	−0.025 (0.876)	−0.049 (0.024)	−0.04 (0.056)	−0.052 (0.012)
Landscape pattern	0.17 (0.005)	0.20 (0.003)	0.173 (0.009)	0.012 (0.755)	0.13 (0.018)	0.06 (0.001)	0.147 (0.007)

**Table 5:** Effects of residents' socioeconomic attributes, use characteristic and physical environment of green spaces on the total level of CES with the multivariate linear regression analysis.

**Variable**	**Model 1**		**Model 2**		**Model 3**		**Model 4**		**Model 5**	
	**Beta (CI)**	***P***	**Beta (CI)**	***P***	**Beta (CI)**	***P***	**Beta (CI)**	***P***	**Beta (CI)**	***P***
**OBJECTIVE PHYSICAL ENVIRONMENT**										
Real vegetation coverage ratio	0.25 (0,0.05)	0.048	0.19 (−0.01,0.05)	0.169	0.21 (−0.01, 0.05)	0.152	0.22 (−0.01, 0.05)	0.154	0.21 (−0.01, 0.05)	0.171
Existence of water body					−0.07 (−0.63, 0.37)	0.598	−0.1 (−0.78, 0.43)	0.552	−0.09 (−0.79, 0.45)	0.577
Number of spaces for public activities							0.04 (−0.05, 0.07)	0.767	0.04 (−0.05, 0.07)	0.777
management level									−0.04 (−0.41, 0.34)	0.8437
**SUBJECTIVE PHYSICAL ENVIRONMENT**										
Landscape pattern	0.28 (0.02, 0.18)	0.015	0.24 (0, 0.17)	0.061	0.24 (−0.01, 0.17)	0.065	0.24 (−0.01, 0.18)	0.071	0.25 (−0.01, 0.19)	0.089
Plant decoration	0.26 (0, 0.06)	0.053	0.28 (0, 0.06)	0.045	0.28 (0, 0.06)	0.05	0.27 (−0.003, 0.06)	0.078	0.27 (−0.004, 0.06)	0.085
Coverage of greenspaces			0.13 (−0.02, 0.04)	0.436	0.14 (−0.02, 0.04)	0.41	0.13 (−0.02, 0.04)	0.474	0.14 (−0.02, 0.04)	0.467
Space for public activities	0.28 (0.02, 0.18)	0.015	−0.14 (−0.06, 0.02)	0.297	−0.13 (−0.06, 0.02)	0.359	−0.13 (−0.06, 0.02)	0.372	−0.13 (−0.06, 0.03)	0.379
Occasional visit to park	0.31 (0.01, 0.03)	0.006	0.31 (0.01, 0.03)	0.006	0.32 (0.01, 0.04)	0.007	0.31 (0.01, 0.04)	0.01	0.32 (0, 0.04)	0.019
21–29 years	−0.19 (−0.07, 0.01)	0.088	−0.2 (−0.07, 0)	0.08	−0.23 (−0.08, 0)	0.078	−0.24 (−0.08, 0.01)	0.082	−0.24 (−0.08, 0.01)	0.089
1–2 h	0.21 (0, 0.04)	0.073	0.16 (−0.01, 0.04)	0.237	0.17 (−0.01, 0.04)	0.221	0.17 (−0.01, 0.04)	0.221	0.17 (−0.01, 0.05)	0.226

### Ethics Approval

This study was carried out in accordance with the recommendations of the ethical standards of Henan University of Economics and Law. The protocol was approved by Henan University of Economics and Law. All subjects gave written informed consent in accordance with the Declaration of Helsinki.

## Results

### Social and Economic Characteristics of the Respondents

Table 2 presents the demographic characteristics of the survey respondents and their mean satisfaction with the CES in their residential green spaces. A total of 4,519 residents in 40 residential areas were interviewed. The percentage of female respondents (50.28%) was slightly higher than that of the male ones (49.72%). Most respondents were within the age range of 30–39 years (24.36%), followed by 21–29 (19.7%), <20 (17.1%), 40–49 (15.17%), >60 (13.6%), and 50–59 years (10.07%). In terms of educational attainment, most of the respondents had an undergraduate degree (28.54%) or had completed junior high school or lower (27.73%), junior college (21.3%), or high school (18.05%). The smallest percentage of respondents had a post-graduate degree (4.38%). As for monthly income, respondents with no income represented the largest percentage (31.6%); followed by those earning 3,000–5,000 RMB (29.25%), 1,000–3,000 RMB (25.5%), and 5,000–10,000 RMB (18.55%); and last those earning above 10,000 RMB (5.1%). Almost half of the respondents had lived in the community area for more than 5 years (40.16%), followed by those residing in the area for 1–3 (24.45%) and 3–5 years (22.54%), and last those living in the community for <1 year (14.68%).

### Cultural Ecosystem Services Satisfaction Levels in the Residential Green Spaces

**Figure 2:**
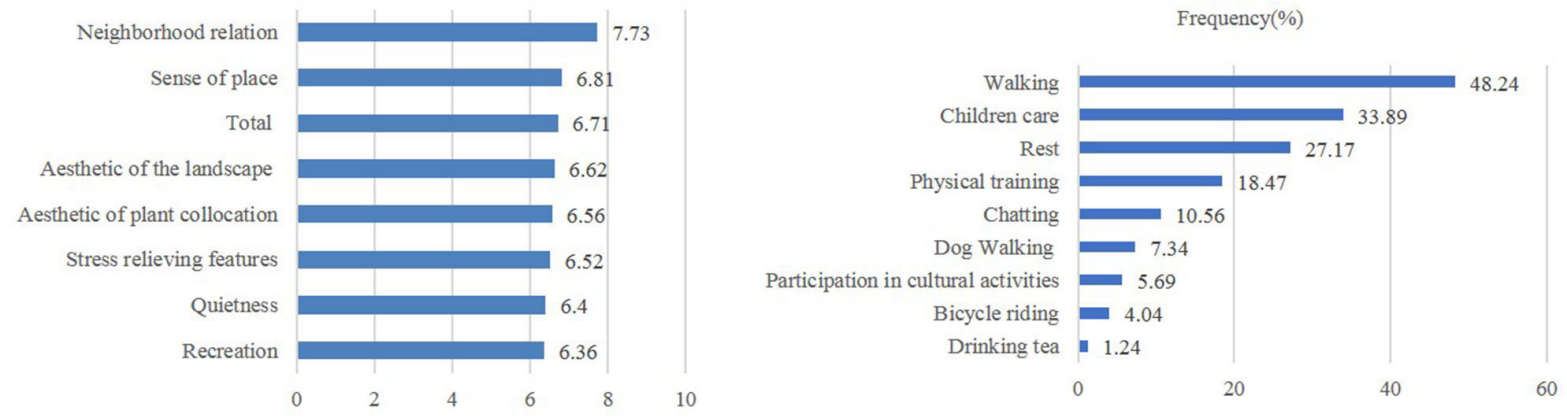
Residents' satisfaction score on cultural ecosystem services (CES) and the main recreational activities in the residential green spaces.

Figure 2 shows the residents' satisfaction level with CES in the residential green spaces, which is based on the respondents' reported usage and satisfaction regarding residential green spaces. Satisfaction with neighborhood relations obtained the highest average score of 7.73 (from a scale of 1 to 10), followed by the sense of belonging (6.81), vegetation landscape aesthetics (6.62), and plant collocation aesthetics (6.56). Satisfaction with recreation services, the quietness of the environment, and stress-relieving features obtained the lowest average scores (6.36, 6.40, and 6.52, respectively). The average overall residents' satisfaction score was 6.71. These results reveal that the residents' satisfaction with various types of CES is relatively similar. The relationship between different CES of green spaces was analyzed using Pearson's correlation (Table 3), which revealed significant positive correlations (*p* < 0.01). The main activities of residents in residential green spaces include walking, childcare, and resting (Figure 2), which accounted for 48.24, 33.89, and 27.17% of the activities, respectively. In addition, residents exercise (18.47%), meet and chat with friends (10.56%), walk their dogs (7.34%), participate in cultural activities (e.g., singing, dancing, calligraphy, playing chess or cards, and painting) (5.69%), ride bicycles (4.04%), and drink tea (1.24%).

Residents frequently visit residential green spaces (Table 2). Approximately 40% of the interviewed residents visit residential green spaces every day, and 30.87% visit at least three times per week. In addition, 11.68% of residents visit residential green spaces at least three times per month, and 18.07% pay occasional visits. However, most residents stay in residential green spaces for a short time: 44.05% stay for nearly half an hour, whereas 37.65% stay for 1–2 h. The proportion of residents staying for a longer time than this is relatively low, with 5.91 and 3.87% staying for 3 and >3 h, respectively. Walking is the major activity of the residents who stay in the residential green spaces for 1–2 h, whereas engaging in social communications and drinking tea are the primary activities of residents who stay for roughly 3 h. Moreover, social communication is the reason why residents stay longer than 3 h.

A statistical analysis of the residents' frequency of communicating with neighbors and friends in the residential green spaces was also performed. The results returned a high overall frequency: 27.95 and 30.71% of the residents chat with others every day and at least three times a week, respectively, and 11.58% chat with other people at least three times every month. Only 17.18 and 12.56% of residents occasionally and hardly chat with neighbors and friends, respectively. In addition, almost half of the respondents pay occasional visits to parks outside the residential areas (43.58%), whereas the lowest proportion visits these parks every day (10.26%). In addition, several people visit parks at least three times a week (24.37%) or at least three times a month (21.79%).

### Determinants of Cultural Ecosystem Services in Residential Green Spaces

According to the univariate linear regression model between the socioeconomic characteristics and residents' satisfaction with CES (Table 4), gender, length of stay, and education background showed no significant correlations. Interestingly, the proportion of people in the age group 21–29 years was negatively correlated with multiple CES, whereas that in the age group 50–59 years was positively correlated with the satisfaction of neighborhood relations and sense of belonging, whereas the age group >60 years was significantly correlated with satisfaction with a quiet environment and a sense of belonging. With respect to income level, residents earning 1,000–3,000 RMB were only slightly satisfied with plant collocation aesthetics.

**Figure 3:**
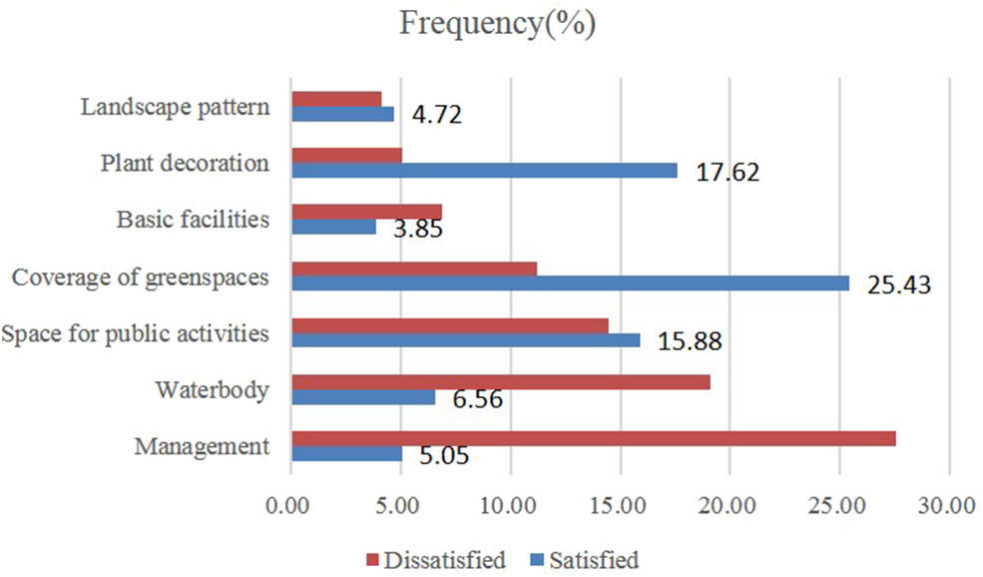
Proportion of respondents who are satisfied and dissatisfied with the quality of residential green spaces.

**Figure 4:**
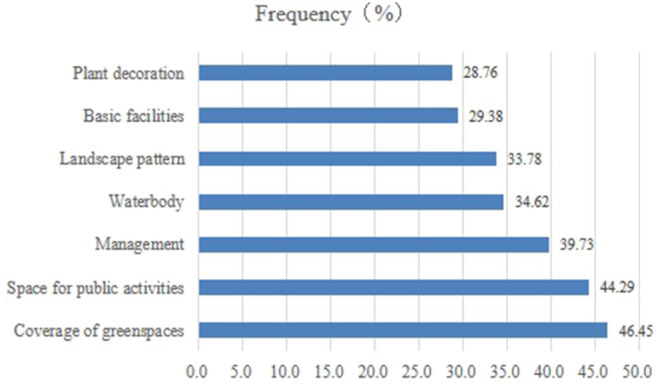
Proportion of respondents who are concerned about the future quality of residential green spaces.

Most residents were dissatisfied with the management, water facilities, and the public activity spaces in the residential green spaces (Figure 3), accounting for 27.53, 19.10, and 14.44% of the total resident population. Moreover, 11.21, 6.89, 5.05, and 4.11% of the residents were not satisfied with the green space coverage, infrastructure, plant collocation, and landscape patterns, respectively. The residents viewed vegetation coverage, public activity spaces, and management as the most important factors that should be considered in the selection of future residential green spaces (Figure 4). The proportions of residents highly concerned with vegetation coverage, public activity spaces, and management were highest, reaching 46.45, 44.29, and 39.73%, respectively. Moreover, those concerned about water facilities, landscape patterns, basic facilities, and plant collocation were 34.62, 33.78, 29.38, and 28.76%, respectively. The basic facilities in residential green spaces include ornamental, artistic, functional, or other equipment for services.

The total level of CES was significantly affected by both the objective and subjective physical environments of the residential green spaces (Table 4). The results from a univariate linear regression showed that the vegetation coverage, management level, number of public activity spaces, and settings of water bodies in residential green spaces were significantly correlated with CES. The proportion of residents who were satisfied with the physical environment, including plant decoration, the coverage of green spaces, the water bodies for public activities, and the landscape patterns of residential green spaces, was significantly correlated with almost all types of CES.

The real vegetation coverage and the proportion of residents who were satisfied with plant decorations and landscape patterns were the only physical environment variables to emerge as significant in the multivariate analysis (Table 5), and their association with the total level of CES was examined after progressive adjustment for different blocks of variables. The proportion of residents who were satisfied with the coverage of green space was removed in the final model; we supposed it could be attributed to the multicollinearity between landscape pattern, plant decoration, and coverage of green spaces. However, the relationship between objective physical environment variables and CES attenuated after adjustment for other variables, indicating that CES was mainly influenced by residents' subjective perception of the physical environment. In addition, the percentage of residents occasionally visiting parks outside the residential areas was also significantly correlated with the level of CES.

## Discussion

### Cultural Ecosystem Services Satisfaction Levels in the Residential Green Spaces

Although some studies have suggested that CES in residential green spaces are less valuable than those in urban green spaces, many researchers have stated that the inherent cultural value does not determine the use frequency of residents and that the distance to green spaces is closely related to individual interests (6, 10, 24, 34, 48, 49). Therefore, evaluating CES will further enrich the study of urban ecosystem services.

We defined the satisfaction level with CES <4 as low, 4–7 as medium, and >7 as high. The total satisfaction level with CES in the residential green spaces in Zhengzhou was medium (6.71), whereas residents' satisfaction with different types of CES varied. Satisfaction with the recreational services obtained the lowest score, which can be attributed to the absence of public activity spaces and facilities in most residential green spaces. Satisfaction with the overall landscape aesthetics of residential green spaces and plant collocation was relatively high, signifying the good aesthetic service level in residential green spaces in Zhengzhou. The level of spiritual services was complicated. Satisfaction with neighborhood relations and sense of belonging was the highest, whereas satisfaction with stress-relieving features and quietness was lower than the previous two factors.

Unlike previous studies, our study evaluated CES by examining residents' satisfaction, which was lowest for recreational services and highest for sense of place and neighborhood relations (Figure 2). Previous studies have highlighted that recreational services in urban green spaces have the highest value among all relevant factors (10, 50), whereas other studies have discovered that aesthetic features are most important (13, 14, 51). In this study, residents' satisfaction with the different CES in residential green spaces was analyzed to evaluate the supply capacity of CES. Gaps between the supply and demand of CES mostly account for the significant difference between our results and other research. However, identification of CES is mainly based on the subjective perception of residents, which produces great variability. Cultural background, customs, social status, and other socioeconomic factors influence people's perception of CES. In addition, the type of green space involved in different research also has an effect.

Multiple CES originate from the natural attributes of urban green spaces. Significantly positive correlations have commonly been observed among the different types of CES (9, 10, 13, 52). However, other studies have also discovered significantly negative correlations (14). Respondents might find it difficult to distinguish between the different types of CES, indicating that various types of CES are concentrated in a specific space (13, 53). This finding implies internal correlation and inseparable natural attributes of the various types of CES and further proves the binding effect of different CES, which might be related to the fact that the different types of CES derive from the natural attributes of the ecosystem (i.e., the surrounding natural environment).

In dense urban regions, CES provided by residential green spaces can stimulate the residents' positive attitude toward neighborhood relations, which could compensate for environmental inequality in the urban area that is, the insufficiency of other popular or large green spaces. Urban parks and woods are the most important among the different types of green spaces (10), providing high social, economic, environmental, and ecological services and values. However, the spatial distribution of these green spaces varies widely in urban areas, thereby contributing to widespread environmental inequities. The relationship between CES and residents' visit frequency to parks outside their communities in our study demonstrated that residential green spaces can effectively compensate for the lack of nearby parks owing to their proximity to residents' living areas. Therefore, the construction, investment, planning, and design of residential green spaces should be paid additional attention.

### Socioeconomic Attributes of Cultural Ecosystem Services in Residential Green Spaces

CES in green spaces are stable and can be directly determined by the green landscape. Initially, we suppose the length of living in a community was believed to have caused polarization of residents' subjective evaluation of CES in green spaces. Residents who live in a community for a long time frequently visit residential green spaces, are more familiar with the surrounding environment than other groups, and thus make extensive subjective evaluations. Moreover, such residents are more socially integrated than residents who have been living in the community for a short time (42). However, residents may eventually become increasingly dissatisfied with the unreasonable characteristics of green spaces, resulting in low satisfaction with the different CES. Conversely, a short time of residence may easily polarize evaluation owing to the freshness of the residential environment. However, no significant correlation between residents' interest in CES and length of residence was observed in the present study, which can be attributed to the stability of the cultural service characteristics of the landscape in the residential green spaces of the 40 residential communities selected. In other words, established CES characteristics are difficult to change once the landscape is formed. Previous studies have reported that the landscapes in urban green spaces play an important role in improving the CES in an ecosystem (51, 54–57). Given this fact, reasonable and scientific planning and design of the landscape become extremely important for the future. Nassauer et al. stated that the ecologically innovative designs of metropolitan residential landscapes were conducive to the enhancement of long-term cultural sustainability (58).

Age is the main influence on satisfaction with CES in residential green spaces in this study. The proportion of residents who were 21–29 years old demonstrated significantly negative correlations with recreation, aesthetics, neighborhood relations, stress-relieving features, and sense of belonging (*p* < 0.001). This finding reveals that these young residents are the least satisfied with the different CES among the other residents of the community. This phenomenon can be attributed to the low satisfaction of this age group with the management and infrastructure of residential green spaces, which are key factors that determine residents' satisfaction with the CES in green spaces. The age group 21–29 years showed significantly negative correlations with the management and infrastructure of green spaces. Moreover, this age group was mainly composed of single individuals. Compared with married residents, single residents are less satisfied with CES and the surrounding residential environment (33, 58, 59). The respondents in the age group >50 years exhibited positive correlation with satisfaction regarding sense of belonging. Previous studies have reported that, compared with other age groups, older people possess a stronger sense of belonging (14) and aesthetic appreciation (60) of the urban environment, which can be attributed to their higher visit frequency to urban green spaces.

The low-income groups in our study demonstrated dissatisfaction with CES. The higher the proportion of residents with a 1,000–3,000 RMB income level, the lower the satisfaction with plant collocation. This result is consistent with the findings of Riechers et al., who discovered that residents with lower incomes had weaker natural cognition, cultural heritage, sense of social belongingness, and satisfaction with urban green spaces than those with higher incomes (14). Several studies have reported that low-income groups, or those with low socioeconomic status, were more frustrated than high-income individuals (28, 61), which may affect their satisfaction with the surrounding green spaces and thus causes negative impacts on their health. Moreover, studies have proven that income level is positively related to interpersonal relationships and the physiological and psychological health of residents (28). However, the present study determined that the gender and cultural level of residents had no significant impact on satisfaction with CES in residential green spaces.

### Cultural Ecosystem Services and Visit Duration in Residential Green Spaces

Urban residents frequently visit residential green spaces; hence, CES in the green spaces within residential areas cannot be ignored. In this study, ~40% of residents visited residential green spaces every day. The visit frequency of residents has been a focus of studies on CES in urban green spaces. The higher the visit frequency, the higher the CES level in the green spaces, which is because visiting green spaces is conducive to physiological and psychological health (29, 42, 62, 63). Many studies have measured the CES level in green spaces using the visit frequency of urban residents (9, 48, 53, 58, 64). This study discovered that the visit frequency of residents showed no significant effect on their satisfaction with CES. This result is consistent with other studies, in which the visit frequency of residents to urban green spaces was discovered to have no significant correlation with the CES value (10), satisfaction with green spaces (6), or people's psychological health (47). Subjective evaluation of residents' demand for CES emphasizes the attributes of green spaces, such as their type, area, distance, and landscape pattern, whereas the evaluation of CES supply is influenced not only by the physical characteristics of green spaces but also by the individual differences of residents, such as age (14), individual emotional factors (6), social group (65), and even survey research methods (6). In this study, respondents belonging to the age group 21–29 years only occasionally visit green spaces. These respondents showed the lowest satisfaction with the aesthetics of the green spaces, which explains the correlation between visit frequency and CES.

Visit time, especially of 1–2 h of duration, is important in the investigation of the satisfaction with CES in green spaces. The proportion of residents in this study staying in the residential green spaces for this duration was high, and the satisfaction of these residents with the recreational services and stress-relieving features was proportionally high. Previous study have revealed that the visit time of residents and the flow duration of cultural services in the residential green spaces last for 1–2 h (6), and we also found that the proportion of residents visiting green spaces for 1–2 h was positively correlated with the percentage of residents walking (Table 4). Walking was the main activity of residents staying for this duration, and this activity can greatly improve the physiological and psychological health of residents. However, many roads in residential areas have mixed purposes that include sidewalks, car lanes, facilities for bicycles and electric bicycles, and private car parking lots. In the 10 residential areas with the highest proportion of walking, six areas implement a sidewalk–car lane separated system. Although the remaining four areas adopt a sidewalk–car lane mixed system, large gardens or clustered green spaces hinder walking activities. Therefore, future planning and design of residential green spaces should create landscapes that are appropriate and safe for walking (e.g., designated sidewalks). If the green spaces in the community are limited, then public activity spaces should be enlarged to meet the residents' demands for walking activities.

### Effects of Vegetation Coverage in Residential Green Spaces on Cultural Ecosystem Services

**Figure 5:**
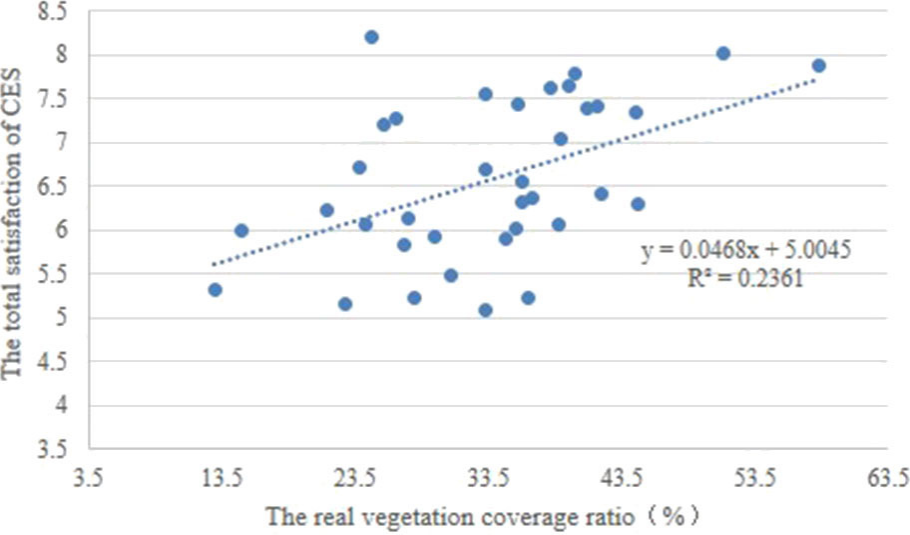
Correlation between the total satisfaction on cultural ecosystem services (CES) and the real vegetation coverage.

**Figure 6:**
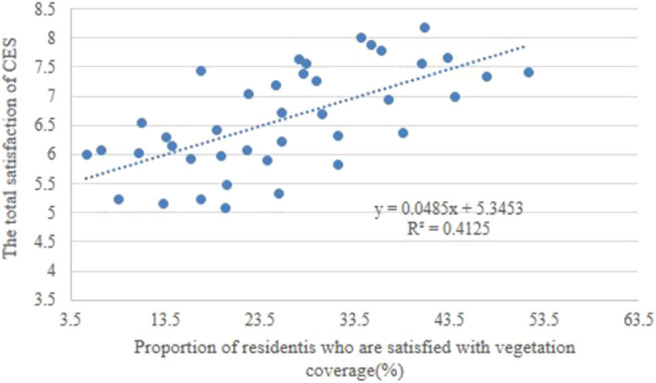
Correlation between the total satisfaction on cultural ecosystem services (CES) and the proportion of residents who are satisfied with residential vegetation coverage.

CES can be improved by increasing green coverage in residential green spaces. Increasing the area of urban green spaces can effectively optimize the biodiversity and carbon fixation of soils (66), regulate the urban microclimate, and reduce surface runoff. However, few studies have focused on the relationship between urban vegetation coverage and CES. Given similar demographic conditions, socioeconomic factors, and living conditions, residents have been found to be happier in larger surrounding green spaces and more satisfied with the surrounding environment than in smaller ones (67). The size of urban green spaces may directly influence their popularity (16). In this study, both objective and subjective green coverage were significantly related to total satisfaction with CES (Figures 5, 6). This inference can explain why residents show great concern with the green coverage in residential communities (Figure 4). Natural vegetation is the source of CES that can increase spiritual and aesthetic services. High green coverage can provide many chances for residents to engage with nature and provide space for water bodies, in particular. In addition, public activity spaces are frequently used in residential communities to stimulate the residents' recreational activities: our study found that both the number and diversity of public activity spaces were higher in residential communities with high green coverage than in those with low green coverage.

With the increase in urban populations and the growing need for housing, urban residential areas are often dominated by high-density communities at the expense of green space. Hence, increasing CES and improving the living environment of residents in a limited green space are an important issue that must be addressed. We found that both residents' perception of plant decoration and landscape patterns in the residential green spaces could directly increase CES (Tables 4, 5). Several studies have suggested that vegetation characteristics (e.g., diversity, vegetation types, abundance, color, new species, morphology, density, and configuration structure) (7, 16, 37), spatial structure, and layout of urban green spaces (6) can influence CES. The close-to-nature attribute of green spaces has been widely accepted as an effective means to improve urban green spaces (9, 16, 37). Moreover, numerous studies have proved that the quality of green spaces is extremely important in improving the physiological and psychological health of residents (47, 68). In this study, residential communities with high green coverage and large lawn areas are popular with residents owing to their accessibility and aesthetics. By contrast, dense shrub vegetation is not conducive to the improvement of green space CES owing to non-accessibility and the possibility of mosquito infestation. In Zhengzhou, half of the residential communities with high green coverage are dominated by dense shrub, which may be due to the low management cost, easy pruning, and accessible irrigation. Therefore, we recommend the cultivation of trees is economical and practical in dense urban residential areas. For communities with low green coverage, the lack of CES can be compensated for by increasing the public activity spaces.

### Perception of Infrastructure and Management as the Key Influencing Factors of Cultural Ecosystem Services in Green Spaces

**Figure 7:**
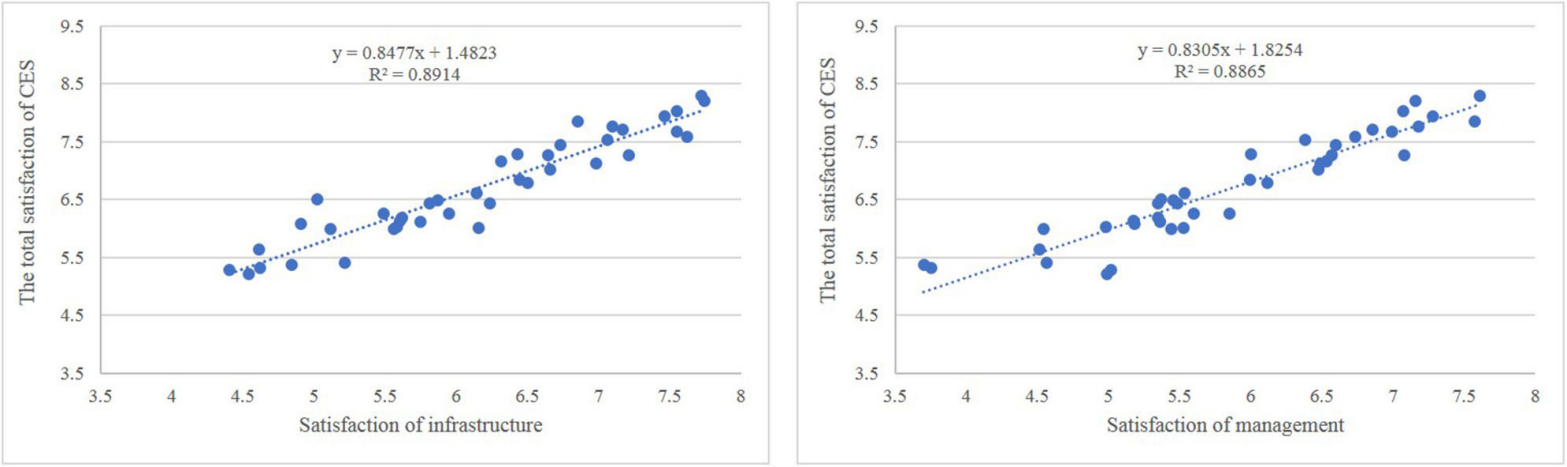
Correlation between the satisfaction on cultural ecosystem services (CES) and satisfaction on management and infrastructure.

Infrastructure in residential green spaces includes public activity spaces (e.g., small squares, gardens, and pavilions), water facilities (e.g., fountains, pools, and artificial lakes), recreation facilities (e.g., fitness and integrated playground equipment), and artistic decorations (e.g., sculptures, chairs, streetlights, and other indicators). The results of this study suggest that satisfaction with the infrastructure in residential green spaces exhibits a significant positive correlation with the overall satisfaction with CES (Figure 7). The positive effects of infrastructure and the convenience of urban green spaces in improving satisfaction with CES have been established by several research studies (10, 13, 34, 59, 69). Furthermore, public activity spaces in residential areas, especially green spaces in squares and gardens, can improve satisfaction with CES. These spaces were the second most important consideration of residents when selecting communities to live in, next to green spaces coverage. Moreover, the water facilities in residential green spaces, which is the fourth most important resident concern, can improve satisfaction with CES in green spaces (Figure 4). Many studies have established the crucial role of wetlands (artificial or natural) in urban green spaces in improving CES (35, 49, 70–72). Plieninger observed that urban residents in Eastern Germany frequently visit water bodies (13) and often give a high evaluation (51). In summary, urban residents highly prefer wetland and water bodies. However, the water facilities in many communities in Zhengzhou are wasted or improperly managed. Therefore, residential green spaces should receive efforts to strengthen the layout and management of water landscapes and water facilities in the future.

**Figure 8:**
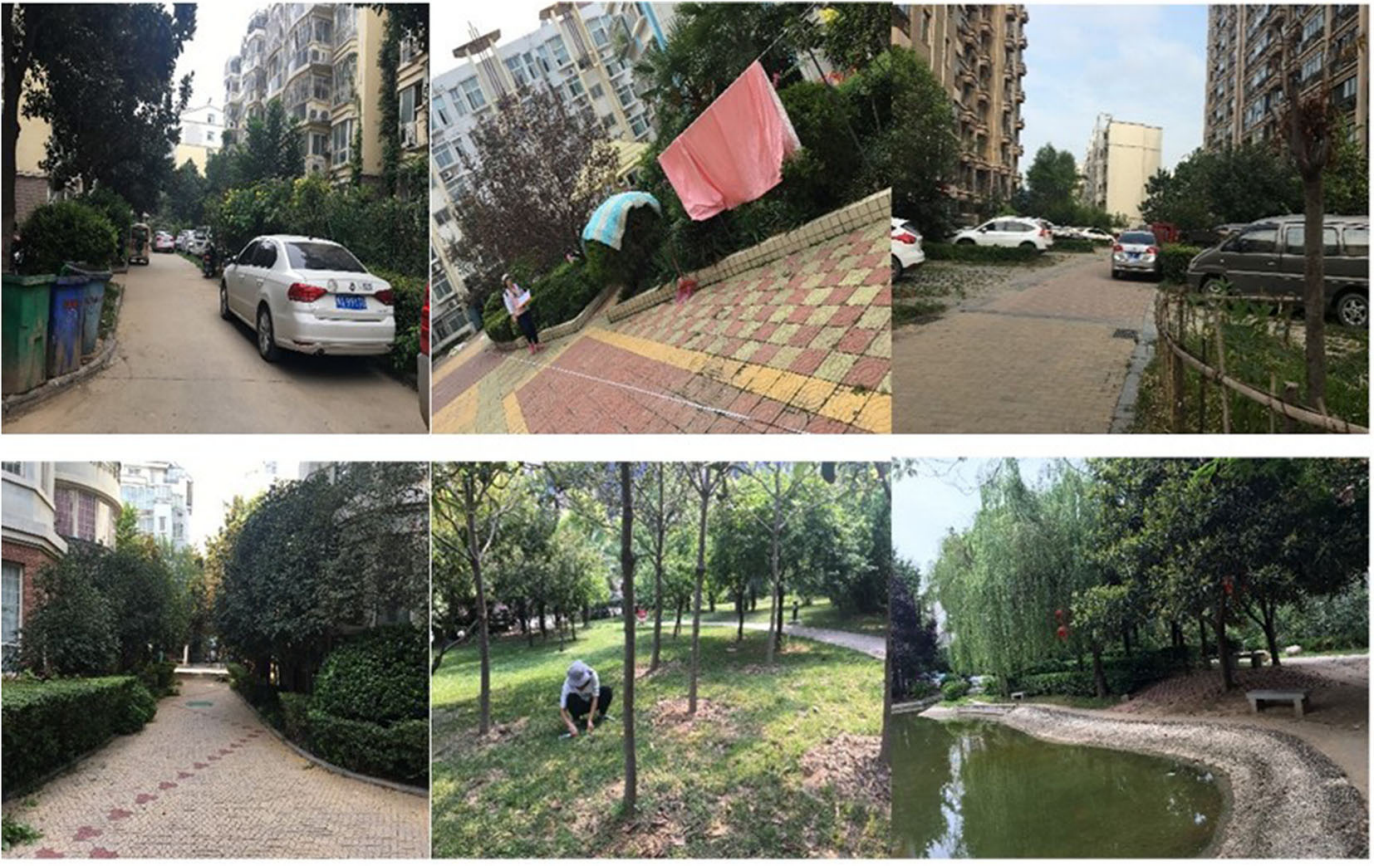
Differences in the management level between the residential communities with the lowest (above) and highest (below) satisfaction on the cultural ecosystem services (CES) in green spaces.

Management of residential green spaces could improve CES greatly in residential green spaces (Figure 7). Many studies have proved that satisfaction with urban residential spaces is closely related to a graceful visual landscape (34, 37). However, the management of green spaces, such as irrigation, clipping, cleaning, and tidying, can directly influence the aesthetic characteristics of green spaces. These characteristics (Figure 8) include (1) cleanliness, which is the top concern of residents (9) and can directly influence the satisfaction with the residential environment (59); (2) standardization, which involves preventing the use of green spaces for other purposes, such as hanging out clothes and providing parking lots for bicycles, electric bicycles, and even motor vehicles; and (3) uneven heights of vegetation, drought events, and weed spreading, which may be present in residential green spaces owing to inadequate daily management (e.g., lack of clipping, irrigating, and weeding). Management was the third concern of residents in residential green spaces (Figure 4): therefore, additional attention and effort should be dedicated to performing regular high-quality maintenance and management of residential green spaces in the future.

### Limitations

Our study has several major limitations. This study only selected five types of CES, namely, leisure and entertainment services, spiritual services, aesthetic services, sense of belonging, and social relations. Other CES (e.g., landscape identity, education, history, religion, and heritage values), which are popularly involved in other research, were not included in our study. The main reason for this exclusion was that the residential areas selected in this study were built only after 2000. We hypothesized that the values of cultural heritage, education, history, and religion would be relatively weak. All 40 residential areas are located in the populated areas of Zhengzhou, which are mainly characterized by dense buildings, as determined by the socioeconomic status levels of developing countries. Future studies should explore the influences of spatial infrastructure arrangements in green spaces on CES, including vegetation type, quantity and area of public activity spaces, type and amount of infrastructure, form and area of water landscapes, and other objective factors. Moreover, landscape and species composition and structure (landscape and vegetation) should also be investigated because such factors could influence residents' contribution to CES in the residential green spaces. The answers to these issues could provide direct scientific references for the landscape planning and design of residential green spaces. Moreover, we chose 40 residential areas within neighboring cells to ensure a sufficient sample size (number of families > 600) and minimize the impact of the surrounding environment on the CES satisfaction of residential green spaces. However, these residential areas include high-rise buildings (>18 floors), mid-rise buildings (7–18 floors), and low-rise buildings (4–6 floors) and are characterized by various types and styles of buildings. These conditions may have affected residents' direct perception and satisfaction with green space landscapes. We suggest that future research focuses on residential areas with consistent socioeconomic levels, including housing prices, architectural styles, green space coverage, management levels, geographical locations, and surrounding green space distribution, to explore the rational arrangement of the green space landscape pattern in a limited space. Such selection will further improve the green space ecosystem services and human well-being and provide a direct theoretical basis for the spatial planning and design of urban green space landscapes.

## Conclusions

Exploring CES in residential green spaces could greatly enrich urban ecosystem services research. The most important research is to clarify the relationship between ecosystem services and human well-being. In this study, we found that walking, childcare, and resting were the most common recreational activities of residents. The results of the analysis show that satisfaction with recreational services in the residential green spaces was the lowest (6.26, 1–10), which can be attributed to the absence of public activity spaces. In contrast, satisfaction with neighborhood relations and the sense of place was the highest at 7.73 and 6.81, respectively, followed by aesthetic services (6.59), indicating that the spiritual and aesthetic services in the residential green spaces are excellent. Age and income status can influence residents' satisfaction with CES: young individuals (21–29 years old) expressed the lowest satisfaction with residential green spaces than did other groups, which might be influenced by their single status and their low satisfaction with the infrastructure in the residential environment.

Satisfaction with CES significantly increased with vegetation coverage, indicating that green vegetation is a source of high CES satisfaction. Compared with other factors, high green coverage is mostly preferred by residents. In addition, public activity spaces, management, infrastructure, and water landscapes are the other key influencing factors of CES satisfaction. Therefore, to maximize CES in residential green spaces, we suggest that public activity spaces should be increased and the daily management of residential areas should be improved when green coverage is limited. Moreover, basic facilities, particularly water landscapes, should be encouraged during the planning and design of residential green spaces. These steps are more effective and realistic in improving the CES of green spaces within areas of dense building density than increasing green space areas.

We suggest that the subjective indicators perceived by residents contribute more to CES than the objective physical environment of residential green spaces. The main reason is that CES refers to human well-being provided by green spaces, which implies residents' demand for green spaces. Future research on the relationship between green spaces' characteristics and CES should consider the physical environment (e.g., biodiversity, green space coverage, species matching, and landscape characteristics) preferred by residents, especially the gaps between the actual and the preferred characteristics favored by residents. For example, understanding the socioeconomic attributes of CES could clarify the demand characteristics of different social groups for urban green space. We suggest future research should pay more attention to different social groups' diverse demands of CES, for example, the use characteristics of the different types of urban green spaces and the diverse landscapes of the same green type. Such consideration will help ameliorate the existing planning and management of urban green spaces, maximize CES, and then protect human health. We suggest the future evaluation of urban green spaces should combine the residents' perception of demand and supply of CES, clarify the gap and trade-off between them, and then determine the key elements that affect the demand of residents, which is the fundamental purpose of urban ecosystem service research. We suggest that the answers to the above research questions will help provide constructive suggestions for building a multifunctional urban green space landscape, which is a path to urban environmental equality and a sustainable urban landscape.

## Data Availability Statement

The raw data supporting the conclusions of this article will be available from the corresponding author upon reasonable request.

## Author Contributions

All authors listed have made a substantial, direct and intellectual contribution to the work, and approved it for publication.

## Conflict of Interest

The authors declare that the research was conducted in the absence of any commercial or financial relationships that could be construed as a potential conflict of interest.
