# Supplementary material for: Evaluating Cultural Ecosystem Services of Urban Residential Green Spaces From the Perspective of Residents' Satisfaction With Green Space
Source: Front Public Health. 2020 Jul 17;8:226. doi: 10.3389/fpubh.2020.00226 (PMC7379909; doi:10.3389/fpubh.2020.00226)
Supplement: Supplementary file 1 [file Data_Sheet_1.docx]

**Appendix 1 Questionnaire on Cultural Ecosystem Services of green spaces in Residential Communities of Zhengzhou City**

Time Place Investigator

1.Your sex is： A. male B. female

2. Length of your stay in your residential community：

A.<1year B.1-3 years C.3-5 years D. ＞5 years

3.Your age is:

A. <20 years old B.21-29years old C.30-39 years old D.40-49 years old

E.50 to 59 years old F.>60 years old

4.Your educational attainment is:

A.junior middle school and below B.high school or technical secondary school C junior college D.undergraduate E. graduate

5.Your occupation is:

A.clerks B.workers C.peasants D.teachers E.students F.freelancers G. technicians,middle-level cadres,managers H.entrepreneurs,company owners I.retirees J.unemployed persons

6.Your income is:

A.no income B.1000-3000 C.3000-5000 D.5000-10000 E.>10000

7.The frequency of visiting green spaces in residential community:

A.everyday B.three or more times a week C.three or more times a month D. once in a while

8.How long do you usually stay in green spaces in your community every day?

A.hardly ever B.about half an hour C.about an hour D.about three hours E.half day and more

9.Your main activities in green spaces are:

A.walking B. resting C. looking after children D.participating in cultural activities (calligraphy, singing, dancing, painting) E.exercising F. meeting friends and chatting G.walking the dog H. riding I.playing cards J. drinking tea

10.Where do you spend most of your time in green spaces?

A.Garden B.Square C. Footpath D.Lawn E.housing greenbelt

11. How satisfied are you with the recreational activities provided by green spaces in your community?

Very dissatisfied 1, 2, 3, 4, 5, 6, 7, 8, 9, 10 Very satisfied

12.Does your community often hold some cultural activities in green spaces such as competitions，folk activities，dance rehearsals?

A. Yes, often B. Yes, occasionally C.No. hardly

13.The cultural activities you are engaged in in green sapces are mainly:

A.Studying and reading newspapers B.Singing songs and dramas, dancing C.Calligraphy D.Fairs and games E.Online Chatting F.no

14.The time you spend on cultural activities in green spaces of your community is:

A.About half an hour B.about an hour C.about two hours D、about three hours E.half day and above F.no

15.How satisfied are you with the cultural and educational services provided by green spaces?

Very dissatisfied 1, 2, 3, 4, 5, 6, 7, 8, 9, 10 Very satisfied

16.How beautiful do you think the overall landscape pattern of green spaces?

Very ugly 1, 2, 3, 4, 5, 6, 7, 8, 9, 10 Very beautiful

17.How beautiful do you think the plant decoration of green spaces

Very ugly 1, 2, 3, 4, 5, 6, 7, 8, 9, 10 Very beautiful

18.Are you allergic to the plants ? What are they?

A.No B.Yes The allergic plant

20.How suited do you think the landscape design of green spaces for quiet visiting?

Very unsuited 1, 2, 3, 4, 5, 6, 7, 8, 9, 10Verysuited

19.Are you usually alone or with others in green spaces?

A. Alone B. with neighbours C. with families D.with friends

20.How often do you communicate with your neighbors or family in the green spaces?

A.Everyday B.three or more times a week C.three or more times a month D.once in a while E.hardly

21.How much do you think green spaces is good for nationhood relationship?

Very bad 1, 2, 3, 4, 5, 6, 7, 8, 9, 10 very well

22.How stressful do you feel in your life?

Very stressful 1, 2, 3, 4, 5, 6, 7, 8, 9, 10 very stress-free

23.How much do you think green space is good for relieving pressure?

Very unsuited 1, 2, 3, 4, 5, 6, 7, 8, 9, 10very suited

24.How much do you like living in your community?

Not satifsfied 1, 2, 3, 4, 5, 6, 7, 8, 9, 10 Very satisfied

25.How much sense of belonging contributed by green spaces?

Very bad 1, 2, 3, 4, 5, 6, 7, 8, 9, 10 very well

26.What are you most satisfied with green space?

A.plant decoration B. vegetation coverage ratio C.water body setting D. public activities space E. Landscape pattern F. facilities G. management H.other else

27.What are you most dissatisfied with the green space of your community?

A.Plant decoration B. vegetation coverage ratio C.water body setting D. public activities space E. Landscape pattern F. facilities G. management H.other else

28.How satisfied are you with infrastructure within green spaces of your community. (recreational facilities, rest facilities, sculptures, fountains, newspaper bars, etc.)

Very unsatisfied 1, 2, 3, 4, 5, 6, 7, 8, 9, 10 Very satisfied

29. How satisfied are you with the management of green sapces?

Very bad 1, 2, 3, 4, 5, 6, 7, 8, 9, 10 Very well

30.How often do you go to parks outside the community each week?

A.everyday B.three or more times a week C.three or more times a month D.once in a while

31.What is the most important for green spaces of residential communities.

A.plant decoration B. vegetation coverage ratio C.water body setting D. public activities space E. Landscape pattern F. facilities G. management H.other else
